# Tonsils are major sites of persistence of SARS-CoV-2 in children

**DOI:** 10.1128/spectrum.01347-23

**Published:** 2023-09-22

**Authors:** Thais Melquiades de Lima, Ronaldo Bragança Martins, Carolina Sponchiado Miura, Maria Vitória Oliveira Souza, Murilo Henrique Anzolini Cassiano, Tamara Silva Rodrigues, Flávio Protásio Veras, Josane de Freitas Sousa, Rogério Gomes, Glaucia Maria de Almeida, Stella Rezende Melo, Gabriela Condé da Silva, Matheus Dias, Carlos Fabiano Capato, Maria Lúcia Silva, Veridiana Ester Dias de Barros Luiz, Lucas Rodrigues Carenzi, Dario Simões Zamboni, Daniel Macedo de Melo Jorge, Fernando de Queiroz Cunha, Edwin Tamashiro, Wilma Terezinha Anselmo-Lima, Fabiana Cardoso Pereira Valera, Eurico Arruda

**Affiliations:** 1 Department of Cellular and Molecular Biology and Pathogenic Bioagents, University of São Paulo School of Medicine, Ribeirão Preto, São Paulo, Brazil; 2 Department of Clinical, Toxicological and Bromatological Analysis, University of São Paulo School of Pharmaceutical Sciences, Ribeirão Preto, São Paulo, Brazil; 3 Department of Ophthalmology, Otorhinolaryngology and Head and Neck Surgery, University of São Paulo School of Medicine, Ribeirão Preto, São Paulo, Brazil; 4 Department of Biochemistry and Immunology, University of São Paulo School of Medicine, Ribeirão Preto, São Paulo, Brazil; 5 Department of BioMolecular Sciences, University of São Paulo School of Pharmaceutical Sciences, Ribeirão Preto, São Paulo, Brazil; 6 Department of Pharmacology, University of Sao Paulo School of Medicine, Ribeirão Preto, São Paulo, Brazil; U.S. Food and Drug Administration, Silver Spring, Maryland, USA

**Keywords:** COVID-19, virus persistence, viral infection, children, persistent infection, palatine tonsil, adenoid, respiratory viruses, pediatric infectious disease, otorhinolaryngology

## Abstract

In the present study, we show that SARS-CoV-2 can infect palatine tonsils, adenoids, and secretions in children without symptoms of COVID-19, with no history of recent upper airway infection. We studied 48 children undergoing tonsillectomy due to snoring/OSA or recurrent tonsillitis between October 2020 and September 2021. Nasal cytobrushes, nasal washes, and tonsillar tissue fragments obtained at surgery were tested by RT-qPCR, immunohistochemistry (IHC), flow cytometry, and neutralization assay. We detected the presence of SARS-CoV-2 in at least one specimen tested in 27% of patients. IHC revealed the presence of the viral nucleoprotein in epithelial surface and in lymphoid cells in both extrafollicular and follicular regions, in adenoids and palatine tonsils. Also, IHC for the SARS-CoV-2 non-structural protein NSP-16 indicated the presence of viral replication in 53.8% of the SARS-CoV-2-infected tissues. Flow cytometry showed that CD20^+^ B lymphocytes were the most infected phenotypes, followed by CD4+ lymphocytes and CD123 dendritic cells, CD8+ T lymphocytes, and CD14+ macrophages. Additionally, IF indicated that infected tonsillar tissues had increased expression of ACE2 and TMPRSS2. NGS sequencing demonstrated the presence of different SARS-CoV-2 variants in tonsils from different tissues. SARS-CoV-2 antigen detection was not restricted to tonsils but was also detected in nasal cells from the olfactory region. Palatine tonsils and adenoids are sites of prolonged RNA presence by SARS-CoV-2 in children, even without COVID-19 symptoms.

**IMPORTANCE:**

This study shows that SRS-CoV-2 of different lineages can infect tonsils and adenoids in one quarter of children undergoing tonsillectomy. These findings bring advancement to the area of SARS-CoV-2 pathogenesis, by showing that tonsils may be sites of prolonged infection, even without evidence of recent COVID-19 symptoms. SARS-CoV-2 infection of B and T lymphocytes, macrophages, and dendritic cells may interfere with the mounting of immune responses in these secondary lymphoid organs. Moreover, the shedding of SARS-CoV-2 RNA in respiratory secretions from silently infected children raises concern about possible diagnostic confusion in the presence of symptoms of acute respiratory infections caused by other etiologies.

## INTRODUCTION

SARS-CoV-2 causes COVID-19, which affects people of all ages worldwide (1). Severe COVID-19 is much less frequent in children and adolescents than in adults (2) for reasons not entirely understood, but lower expressions of ACE2 and TMPRSS2 in children’s respiratory tract are contributing factors (3).

We have previously reported high rates of PCR detection of respiratory viruses, including endemic coronaviruses, in tonsils and adenoids from patients with chronic tonsillar diseases, without a recent history of symptomatic airway infections (4). This finding was later confirmed by other groups (5 – 7), indicating that human tonsils are sites of asymptomatic respiratory virus infections. These previous results prompted us to test whether SARS-CoV-2 was also detected in children’s tonsils during the COVID-19 pandemic. Here we report detection of SARS-CoV-2 RNA and protein in tonsils, nasal cytobrushes, and respiratory secretions from children with tonsillar hypertrophy lacking symptoms of COVID-19 and determination of the types of infected cells.

## MATERIALS AND METHODS

### Study design and sample processing

This cross-sectional study was done from October 2020 to September 2021 at the Otorhinolaryngology Division of the School of Medicine of Ribeirão Preto, University of São Paulo, and enrolled 3- to 11-year-old children undergoing adenotonsillectomy to treat recurrent tonsillitis or obstructive sleep apnea. The Ethics Committee of the Clinical Hospital, Ribeirao Preto Medical School, University of São Paulo, approved the study (number 4.465.408). Due to the scarcity of COVID rapid tests in Brazil at the time of the study, the institutional protocol for COVID-19 screening was clinically oriented, based on a questionnaire applied to parents/guardians on the day of surgery. If the patient presented any symptoms of acute respiratory infection, surgery was delayed, and then the test was performed. In summary, exclusion criteria were symptoms of acute respiratory infections in the month before surgery, cranium facial malformations, genetic syndromes, deposit diseases, immunodeficiencies, and suspected tonsillar cancer. Parents/guardians signed informed consent, and a questionnaire recorded on REDCap (Vanderbilt University, USA) was filled with demographics, comorbidities, indications for tonsillectomy, relevant physical examination findings, and previous exposure to COVID-19.

During surgery, the following samples were obtained: bilateral nasal wash (10 mL of saline solution instilled and immediately aspirated); bilateral cytobrush of the olfactory area under a 0° rigid optical view, with a small brush positioned close to the olfactory fossa rotated 10 times and immediately placed into sterile RPMI medium with 4% antibiotic/antimycotic solution (Gibco), adenoid and palatine tonsil tissues placed in the same medium; and 4 mL of peripheral blood for serology. Samples were transported to the laboratory on ice within 2 h.

Tissue specimens washed in PBS were used to prepare samples for RT-qPCR, virus isolation, TMNC purification, and histology. Tissue pieces of ~0.25 cm^3^ were treated with collagenase type I (100 U/mL) and dispase (0.6 U/mL) (Gibco) for 1 h at 37°C and then passed through a nylon mesh to obtain a cell suspension for (i) adding to medium VTM, consisting of minimum essential medium with 20% FBS and 15% glycerol for viral isolation; (ii) RNA extraction using Trizol; (iii) backup in RNA-later (Invitrogen); and (iv) isolation of TMNCs by Ficoll-Paque, which were suspended in freezing medium (RPMI medium with 20% FBS and 10% DMSO). Samples were stored at −80°C. Another piece of tonsillar tissue was placed in Carnoy’s fixative and embedded in paraffin. Nasal washes and cytobrushes were used to prepare aliquots in VTM and Trizol, and the remainder was used to prepare cell pellets that were spotted onto glass slides, fixed with acetone, and stored at −20°C for immunofluorescence.

### Detection and quantitation of SARS-CoV-2 RNA

SARS-CoV-2 RNA was detected by RT-qPCR with primers and probes for the N2, E genes, and the RNAse-P housekeeping gene (Table 1), according to previously published protocols (8). We used the One-step real-time RT-PCR done on a certified Step-One Plus real-time PCR thermocycler (Applied Biosystems, Foster City, CA, USA), with total nucleic acids extracted by Trizol (Invitrogen, CA, USA), according to the manufacturer’s instructions. Briefly, 100 ng of RNA was used for genome amplification, adding specific primers (20 µM), probe (5 µM), and PCRBIO 1-Step Go RT-PCR Master Mix (PCR Biosystems, Wayne, PA, USA), with the following cycling parameters: 45°C for 20 min and 95°C for 2 min, followed by 40 cycles of 95°C for 5 s and 60°C for 30 s. The defined cut-off point value for determining positivity was a cycle threshold (CT) value of 37 for the N gene and a CT value of 40 for the E gene. Viral RNA copy numbers were determined using a 944-bp amplicon from the nucleocapsid gene cloned in the pTZ57R/T vector, which enabled the making of a quantification curve based on serial decimal dilutions. The coefficient of determination was 0.999, and the efficiency was 91%. Viral RNA loads were plotted with GraphPad Prism 8.4.2 194 software.

**TABLE 1:** Sets of primers and probes for the detection of SARS-CoV-2 by RT-PCR

Gene	Oligonucleotide	Sequence
	Forward	5′-ACAGGTACGTTAATAGTTAATAGCGT-3′
E	Reverse	5′-ATATTGCAGCAGTACGCACACA-3′
	Probe	5′-Fam- ACACTAGCCATCCTTACTGCGCTTCG-BHQ1-3’
	Forward	5′-TTA CAA ACA TTG GCC GCA AA-3′
N2	Reverse	5′-GCG CGA CAT TCC GAA GAA-3′
	Probe	5′-Fam-ACAATTTGCCCCCAGCGCTTCAG-BHQ1-3’
	Forward	5′-AGATTTGGACCTGCGAGCG-3′
*RNAse-P*	Reverse	5′-GAGCGGCTGTCTCCACAAGT-3′
	Probe	5′-FAM–TTCTGACCTGAAGGCTCTGCGCG–BHQ-1–3’

### Immunohistochemistry for SARS-CoV-2 antigen in adenotonsillar tissue

SARS-CoV-2 antigen was detected *in situ* in tissue sections by immunohistochemistry (IHC) standardized using Vero CCL-81 cells infected with SARS-CoV-2 Wuhan lineage (Fig. S1). Tissue sections (3 µm) were tested as previously published (9). Slides were stained with primary monoclonal rabbit antibody anti-SARS-CoV-2 nucleocapsid (Creative Diagnostics #CABT-RMJ1) or with a rabbit anti-SARS-CoV-2 NSP-16 (Abcam, ab284038). The signal detection was done with Biotinylated Goat Anti-Rabbit IgG (H + L) (Abcam) and Streptavidin HRP (ThermoFisher). AEC chromogen (Vector) was used for antigen labeling and hematoxylin for staining cell nuclei (Sigma-Aldrich). Furthermore, the sections were scanned on ScanScope VS120 (Olympus) using 40× magnification.

### Immunofluorescence for ACE2, TMPRSS2, and SARS-CoV-2 S protein in tonsils

Tissue sections were deparaffinized in xylene (Synth), rehydrated in decreasing ethanol concentrations (JTBacker), and blocked in PBS with 0.01% BSA (Gibco). Slides were stained with the primary antibodies rabbit monoclonal anti-SARS-CoV-2 S protein (Invitrogen), goat polyclonal anti-ACE2 (R&D), or mouse monoclonal anti-TMPRSS2 (Millipore). Slides were incubated with secondary antibodies AlexaFluor 488-labeled secondary alpaca anti-mouse IgG antibodies and alpaca anti-rabbit IgG AlexaFluor 594 (both from Jackson ImmunoReseacher). Nuclei were stained with DAPI (Vector), and images were acquired by Axio Observer combined with LSM 780 confocal microscope (Carl Zeiss) at 63× magnification. Ten random fields per sample were analyzed at the *x* and *y* focal planes to measure mean fluorescence intensities of ACE2 and TMPRSS2, as analyzed by ImageJ.

### Immunofluorescence for SARS-CoV-2 N protein in nasal cytobrush samples

Slides were incubated with a permeabilizing/blocking solution of PBS with 0.01% Triton, 1% BSA (Sigma), and 5% goat serum. Slides were washed in PBS and incubated with primary rabbit monoclonal anti-rabbit SARS-CoV-2 nucleocapsid antibody (Creative Diagnostics). After blocking with SuperBlock, slides were incubated with Alexa 594-conjugated goat anti-rabbit antibody (Abcam), nuclei were stained with DAPI (Thermo Fisher), and images were obtained with a confocal fluorescence Leica TCS SP8 microscope (Leica Microsystems).

### Flow cytometry of TMNCs

Frozen purified TMNCs were analyzed by flow cytometry after a 30-min staining at 4°C using antibodies for CD4 (PerCP-Cy5.5), CD8 (PE-Cy7), CD11c (PE-Cy7), CD14 (PerCP), CD20 (PE-Cy7), and CD123 (PerCP-Cy5.5) (BD Pharmingen). Cells were then washed, permeabilized, and fixed with BD Cytofix/Cytoperm. Intracellular SARS-CoV-2 was stained with rabbit anti-SARS-CoV-2 NP antibody, followed by anti-rabbit IgG-APC secondary antibody (BD Pharmingen). TMNCs from tonsils RT-qPCR negative for SARS-CoV-2 were used as negative controls. Cell preparations stained only with the IgG-APC secondary antibody were used for calibration of PE acquisition. Acquisitions were performed in fixed cells in a flow cytometer (BD Accuri C6; BD Biosciences) and then analyzed using FlowJo software (Tree Star).

### Serology assays

COVID-19 IgM/IgG detections in patient’s sera were done using two rapid test kits from Nantong Egens Biotechnology and Genrui Biotech Inc, following the manufacturer’s instructions. Determination of VNT_100_ was done by previously published protocol (10).

### Sequencing of SARS-CoV-2 genomes

SARS-CoV-2 genome sequencing followed the ARTIC nCoV-2019 protocol v3 (https://protocols.io/view/ncov-2019-sequencing-protocol-v3-locost-bh42j8ye) (11) with modifications previously proposed (12). The Ligation Sequencing Kit used in the MinION library preparation was the SQK-LSK-109, and Native Barcoding was done with the NANO-EXPNBD196 Kit (Oxford Nanopore, Oxford, UK). The library was loaded on R9.4 Oxford MinIONflowcells (FLO-MIN106) and sequenced using the MinION Mk1B device.

### Bioinformatic analysis and sequence availability

The pipeline included (i) the ONT MinKNOW software for collection of raw data and quality control and (ii) Guppy (v6.0.1) for high accuracy base calling. Assembly of the high-accuracy base called Fastq files was done by the nCoV-2019 novel coronavirus bioinformatics protocol (https://artic.network/ncov-2019/ncov2019-bioinformatics-sop.html) with Minimap2 (13) and Rampart (14) for genome coverage analysis. The assembled genomes were analyzed with Nextclade v1.14.0 (https://clades.nextstrain.org/) (15) and Pangolin v4.0 (https://github.com/cov-lineages/pangolin) (16) to identify the clade and lineages.

Raw Minion Nanopore reads from the experiment were submitted to the SRA NCBI database under the BioProject ID PRJNA876260 and the following BioSample IDs: SAMN30649069, SAMN30649070, SAMN30649072, SAMN30649075, SAMN30649076, SAMN30649077, SAMN30649078, SAMN30649079, SAMN30649080, and SAMN30649081. Control raw Minion Nanopore reads were submitted to the SRA NCBI database under the BioProject ID PRJNA909758 and the following BioSample ID: SAMN32093249.

### Statistical analysis

Continuous data were analyzed by Student *t* test, One sample *t* test, or Mann-Whitney test, depending on normal distribution. Statistical tests and graph plotting were performed with GraphPad Prism 8.4.2 software.

## RESULTS

### Demographic characteristics

A total of 48 patients were enrolled in the study. The patients were aged 3 to 11 years (mean 5.9 ± 2.2); 30 were boys (62.5%), and 24 (50%) did not have associated diseases (Table 2). Among the reported comorbidities, allergic rhinitis was reported in 13 children (27.09%), recurrent otitis media in 3 (6.25%), mild asthma in 2 (4.16%), and two diseases associated in 6 (12.5%). According to the parents/guardians, the last acute upper airways infection requiring or not antibiotics occurred 1 to 24 (average 9.2) months before surgery. Eight children (16.67%) had been exposed to confirmed COVID-19 in the household 1 to 13 months before surgery. Two patients had previous laboratory-confirmed SARS-CoV-2 infection in 3 to 5 months before surgery (Table 2). The complete patient demographic data and the results of the molecular and immunostaining assays for all patients are in Table S1.

**TABLE 2:** Demographic data from the patients enrolled in this study

Characteristics	Number of patients (%)
Sex	
Male	30 (62.5%)
Female	18 (37.5%)
Age	
3 to <6	23 (47.9%)
6 to <9	17 (35.4%)
9 to <12	8 (16.7%)
Surgical indication	
Symptoms suggestive of OSA	27 (56.3%)
Recurrent tonsillitis	8 (16.7%)
Both	13 (27%)
Associated disease	
Allergic rhinitis	19 (39.6%)
Recurrent otitis media	6 (12.5%)
Asthma	4 (8.3%)
None	24 (50%)
Previous diagnosis of COVID-19	2 (4.1%)
Previous contact with infected people	8 (16.7%)

### Detection of SARS-CoV-2 RNA and antigen

**Fig 1:**
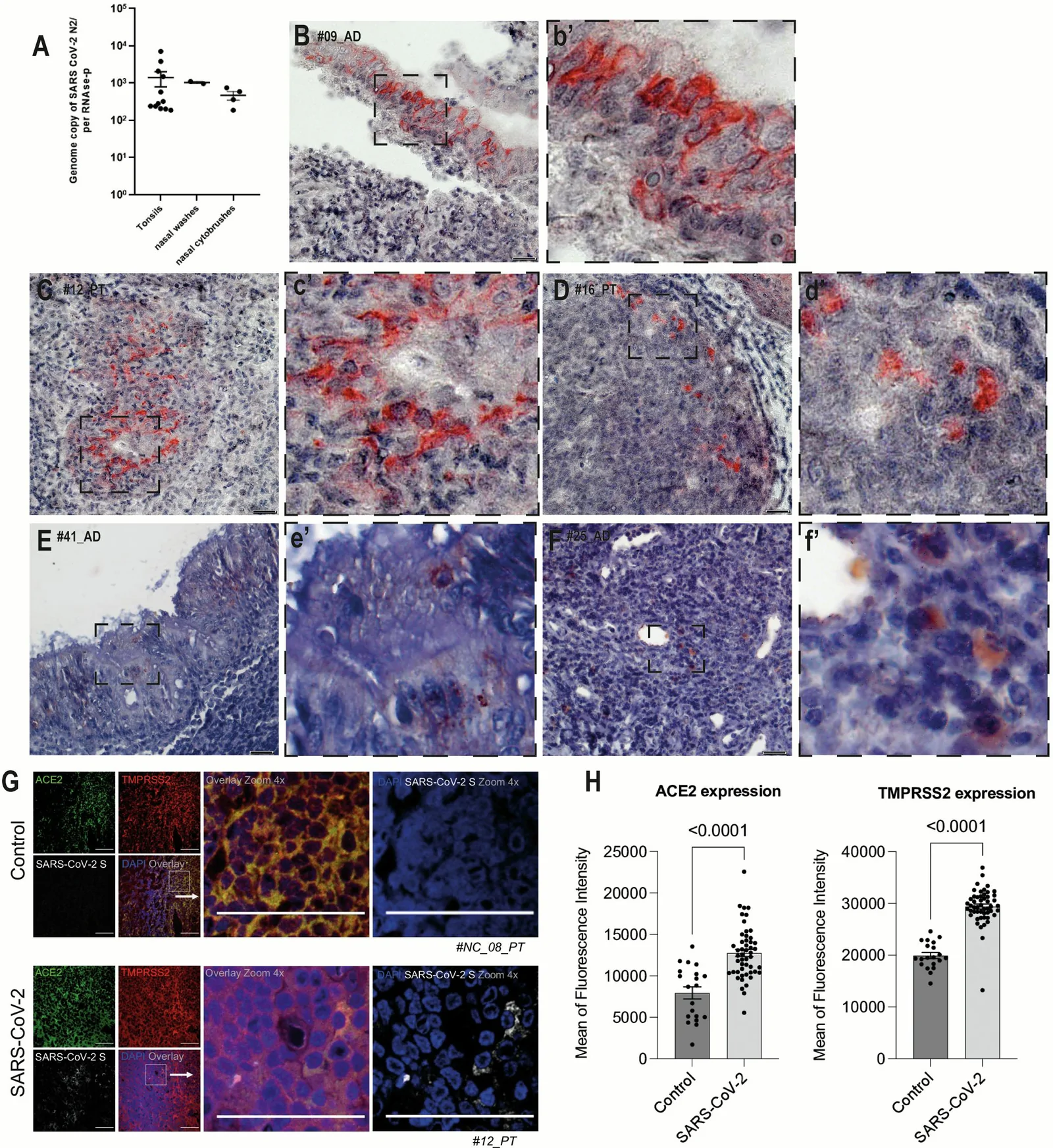
SARS-CoV-2 viral loads and antigen detection. (**A**) Quantification of SARS-CoV-2 RNA genome copies in palatine tonsil and adenoid tissues, nasal washes, and nasal cytological brushes from patients with chronic adenotonsillar disease (mean ± standard deviation). (**B**) Representative section of an adenoid with positive staining for SARS-CoV-2 NP in the pseudo-stratified ciliated epithelium. (**b’**) Higher magnification of the inset (dashed square) in panel B. (**C**) Representative section of a palatine tonsil with positive staining for SARS-CoV-2 NP in the inter-follicular area. (**c’**) Higher magnification of the inset (dashed square) in panel C. (**D**) Representative section of palatine tonsil with positive staining for SARS-CoV-2 NP in follicular lymphoid cells. (**d’**). Higher magnification of the inset (dashed square) in panel D. (**E**) Representative section of an adenoid with positive staining for SARS-CoV-2 NSP-16 in cells in the epithelial region. (**e’**). Higher magnification of the inset (dashed square) in panel E. (**F**) Representative section of a palatine tonsil with positive staining for SARS-CoV-2 NSP-16 in cells in the inter-follicular area. (**f’**). Higher magnification of the inset (dashed square) in panel F. (**G**) Fluorescent staining of palatine tonsil section for ACE2 (green), TMPRSS2 (red), and the S protein of SARS-CoV-2 (gray). (**H**) Mean fluorescence intensity for ACE2 and TMPRSS2 in SARS-CoV-2-infected and control tonsils. Scale bar: 100 µm.

SARS-CoV-2 RNA was detected by RT-qPCR in at least one sample from 13 of the 48 patients (27%). In the majority of them, more than one sample was positive (Table S1). The detection rates of SARS-CoV-2 in our study were as follows: 40% in palatine tonsils, 28% in adenoids, 20% in nasal cytobrushes, and 12% in nasal washes. It is worth noting that the viral loads of SARS-CoV-2 exhibited significant variability, ranging from 186 to 7,114 copies of genome equivalents per copies of endogenous control RNase-P, considering tonsillar tissues, nasal washes, and nasal cytobrushes (Fig. 1A), and although the median viral load in tonsillar tissues was about twofold higher than in nasal specimens, there was no significant difference among samples.

SARS-CoV-2-positive samples were detected in four patients with previous history in the family 1 to 6 months before surgery, and one patient had a previous diagnosis of COVID-19 from 5 months before surgery. Six of 13 SARS-CoV-2-positive children had no comorbidities, and the most frequent comorbidity was allergic rhinitis (5 of 13) (Table S1).

Immunohistochemistry was performed on tissue sections obtained from SARS-CoV-2-positive tonsils, revealing that a significant majority of patients (11 of 13; 84.6%) exhibited positive results for SARS-CoV-2 antigen. Representative images revealed that antigen was detected not only in the tonsillar epithelia but also in scattered cells within the lymphoid compartment, including lymphoid follicles and extrafollicular areas, in both types of tonsils (Fig. 1B, C, and D). Moreover, in addition to detecting the nucleoprotein of SARS-CoV-2, we also observed *in situ* positivity for the non-structural protein NSP16 of SARS-CoV-2 in 53.8% (7/13), a strong indication of viral replication in the lymphoid tissues (Fig. 1E and F).

### Expression of ACE2 and TMPRSS2 in tonsils

Immunofluorescence revealed that the expressions of the main ACE2 and TMPRSS2 in tonsillar tissue sections were significantly more intense in tissues positive for SARS-CoV-2 as compared to negative ones (Fig. 1G). The same areas of enhanced expression were also positive for the SARS-CoV-2 spike protein. The mean differences in fluorescence intensity between SARS-CoV-2-infected and non-infected patients were 4,815 ± 840.8 for ACE2 and 9,471 ± 847.5 for TMPRSS2 (*P* < 0.0001) (Fig. 1H). Other tissues positive and negative from different patients showed ACE2, TMPRSS2, and SARS-CoV-2 spike (Fig. S2).

### Cells infected by SARS-CoV-2 in tonsillar tissues

**Fig 2:**
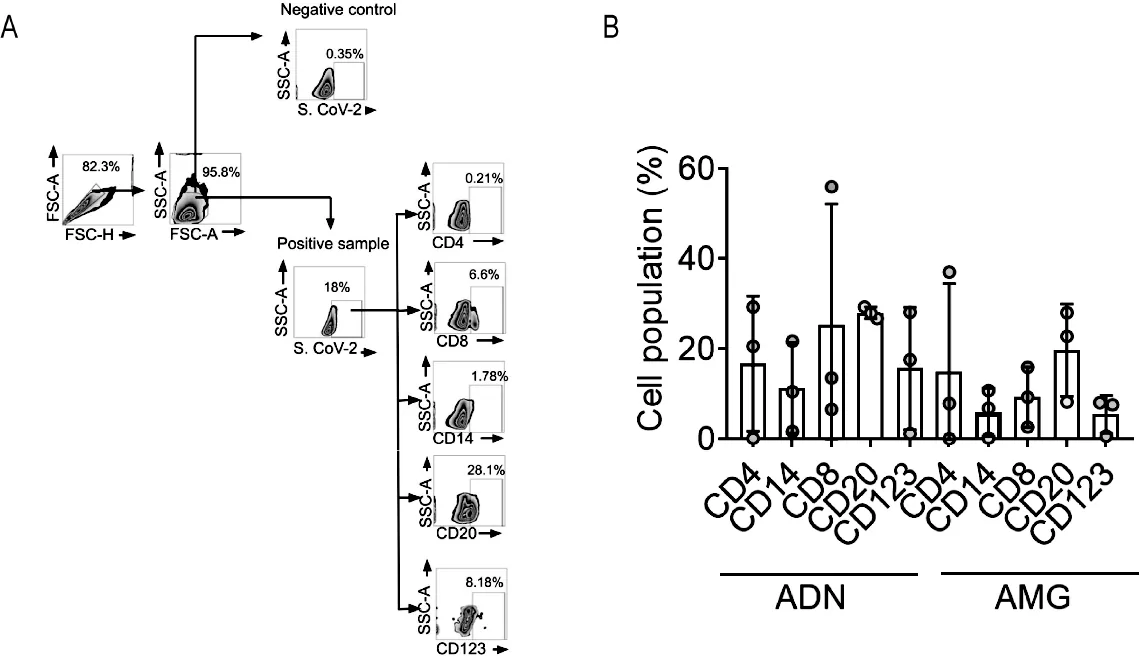
Immune phenotyping of SARS-CoV-2 NP-positive tonsillar cells by flow cytometry. (**A**) Representative gating illustrating the infected population in SARS-CoV-2-negative (Control) and SARS-CoV-2-positive (Patient) tonsils. (**B**) Frequencies of infected immune cells in adenoids and palatine tonsils positive for SARS CoV-2 NP (±SEM). Each circle represents one subject donor of tonsillar tissue.

Gating analyses showed that CD20^+^B lymphocytes were the most frequent SARS-CoV-2-infected cell types, present in median percentages of 22.9% and 28.1% of SARS-CoV-2-infected TMNCs, respectively, in palatine tonsils and adenoids (Fig. 2A). CD4^+^T lymphocytes averaged 7.95% and 20.7% of SARS-CoV-2-positive cells, respectively, in palatine tonsils and adenoids, and CD123^+^ dendritic cells averaged 7.69% and 17.7% of SARS-CoV-2-positive cells, respectively, in palatine tonsils and adenoids, followed by CD8^+^T lymphocytes representing 9.38% and 13.6% SARS-CoV-2-positive cells, respectively, in palatine tonsils and adenoids. At the same time, CD14^+^ macrophages were the least abundant cell type, with medians of 6.9% and 10.6% of SARS-CoV-2-positive cells, respectively, in palatine tonsils and adenoids (Fig. 2B).

### SARS-CoV-2 RNA and antigen detection in nasal cytobrushes

**Fig 3:**
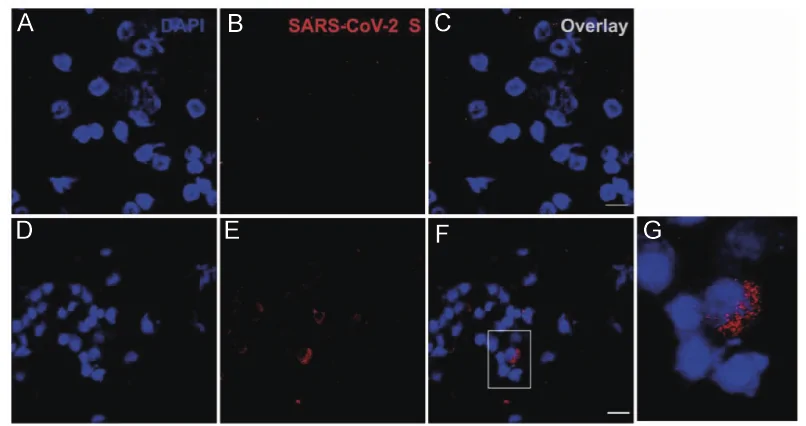
Immunofluorescence for SARS-CoV-2 in cytobrush preparations. Representative fields of cytobrush preparations from SARS-CoV-2-negative (**A–C**) and SARS-CoV-2-positive children (**D–G**), showing positivity for NP protein in some cells. The inset on F is enlarged on G. Scale bar: 25 µm.

Cytobrush samples from the olfactory region were positive for SARS-CoV-2 RNA in 5 of the 13 SARS-CoV-2-positive patients, all of whom were also positive in tonsillar tissues. Immunofluorescence revealed SARS-CoV-2-positive cells in cytobrushes from two of these five patients (Fig. 3).

### SARS-CoV-2 serology

Rapid tests for anti-SARS-CoV-2 IgM and IgG antibodies were done in 12 of the 13 SARS-CoV-2-positive patients, whose sera were available, and IgG was detected in five patients (41.67), while no patient was IgM positive. This indicates that at least 5 of the 13 SARS-CoV-2-positive patients were not in the acute phase of the infection. Sera from all five IgG-positive patients neutralized SARS-CoV-2 *in vitro*, yet in low titers, as determined by VNT_100_ (Table S1).

### SARS-CoV-2 genome sequences

**Fig 4:**
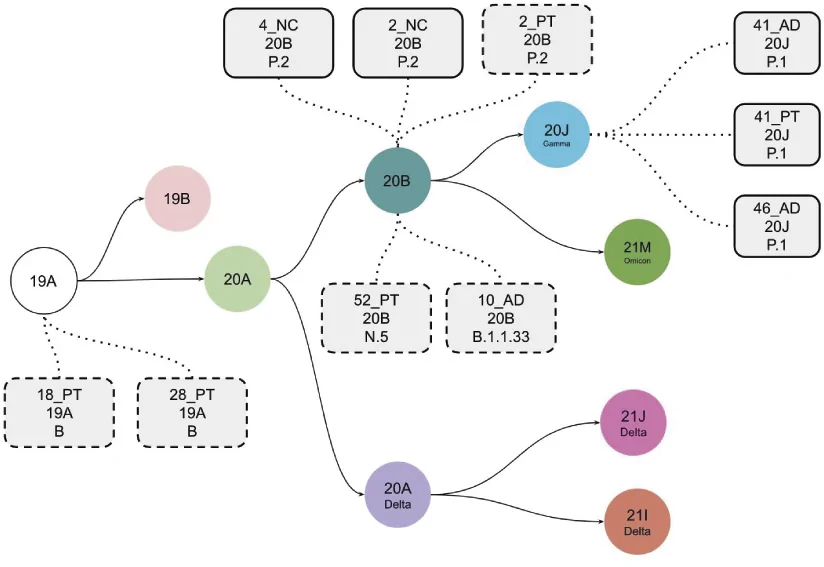
Schematic representation of the evolution of SARS-CoV-2 clades. Each clade is represented by a color circle, and the samples sequenced in the present cohort with the mutational patterns represented by gray rectangles containing the following information, from top to bottom: sample ID, the clade, and the Pangolin lineage assigned by NextClade. The solid rectangles represent SARS-CoV-2 genomes assembled with higher coverage, and the dashed ones represent those with lower coverage but carrying enough defining mutations to enable lineage assignment (AD, adenoid; PT, palatine tonsil; NC, nasal cytobrush).

Nanopore sequencing done in 12 samples (9 tonsils and 3 cytobrushes) from 10 patients yielded SARS-CoV-2 sequences in 8 children (10 samples). The total length of SARS-CoV-2 sequences varied from 346 to 27,615 nucleotides, with six of them covering less than 40% of the genome length, at a 20× depth (Table S1). The sequence analysis revealed several Pangolin lineages (Fig. 4), all of which circulated in Brazil during the study period (17). The results of the pileup analysis for all sequenced samples are shown in Fig. S3. This figure showcases key parameters such as read coverage, base quality, and base percentage.

## DISCUSSION

This study showed that SARS-CoV-2 was detected in upper respiratory tract samples from one-quarter of children undergoing tonsillectomy, even in the absence of recent history of COVID-19. This roughly fivefold higher rate than the approximately 5% reported for seasonal coronaviruses in similar cohorts (4 – 7) may result from the sheer intense circulation of SARS-CoV-2 in Brazil in 2021 or from an enhanced propensity of SARS-CoV-2 to infect tonsils or both.

The time of the patient’s initial exposure to SARS-CoV-2 could not be determined in this cohort nor was it possible to define a past episode of acute infection for most of the SARS-CoV-2-positive children. Thus, these children can be regarded as asymptomatic SARS-CoV-2 carriers, in agreement with reports that children are more likely than adults to have mild or asymptomatic SARS-CoV-2 infections (18, 19). The lower severity of COVID-19 in children can be attributed, at least in part, to some degree of cross-protection afforded by memory T-cell responses to previous infections by endemic coronaviruses (20). Also, a more vigorous innate immune response to SARS-CoV-2 in children than in adults could more efficiently contain the agent at the portal of entry, curb the spread to other tissues, and reduce illness severity (21).

SARS-CoV-2 RNA was detected by RT-qPCR in more than one sample from some of the virus-positive children, with viral loads varying from hundreds to thousands of copies per copies of RNase-p, suggesting that they may have undergone tonsillectomy at different times post-infection. Nevertheless, the time of initial exposure to SARS-CoV-2 was unknown, and most children had no clear symptomatic phase, which hampers the establishment of correlations between duration of infection and viral loads at the time of tonsillectomy. It is important to stress that 5 of the 13 children were IgG positive, but none was IgM positive for SARS-CoV-2.

In addition to the detection of the viral RNA, which could be regarded as some remnant from a past infection, the present study revealed structural viral protein *in situ* in adenoids and palatine tonsils, in both epithelial and lymphomononuclear cells of different lymphoid compartments. This novel information provides further evidence for the presence of viral protein synthesis, hence viral activity, in tonsils of children without overt COVID-19. Furthermore, we successfully detected NSP-16, a robust indicator of SARS-CoV-2 replication. NSP-16 plays a crucial role in the process of viral RNA capping, which is a vital step for efficient viral replication (22). The identification of NSP-16 in lymphoid tissues provides compelling evidence for the ongoing replication of SARS-CoV-2. Also, the presence of SARS-CoV-2 protein in cells from the olfactory region in three children, who also had virus detected in their tonsils, indicates that prolonged SARS-CoV-2 infection is not restricted to tonsillar cells.

SARS-CoV-2 antigen was detected by flow cytometry in the major types of TMNCs, including B and T lymphocytes, macrophages, and dendritic cells. Remarkably, this agrees with our previous report that SARS-CoV-2 infects the same range of PBMC from adult COVID-19 patients, inducing apoptosis of infected cells, thus contributing to lymphopenia (8, 23). Also, post-mortem studies revealed SARS-CoV-2 infection in human lymphomononuclear cells, with histological alterations in spleens, lymph nodes, and gut-associated lymphoid tissue (24, 25). Those findings and the present observations in asymptomatic children suggest that SARS-CoV-2-infected lymphomononuclear cells may intermigrate among secondary lymphoid organs, where intense B lymphocyte maturation and T lymphocyte activation take place.

ACE2 and TMPRSS2 proteins are highly expressed in the upper respiratory tract (26), and the present finding of even higher ACE2 and TMPRSS2 expressions in SARS-CoV-2-infected tonsils may suggest that SARS-CoV-2 tonsillar infection promotes increased expression of ACE2 and TMPRSS2. Alternatively, a higher constitutive expression of ACE2 and TMPRSS2, depending on individual variation, could predispose some children to SARS-CoV-2 infection in tonsils.

B lymphocytes comprised roughly one-quarter of SARS-CoV-2-infected TMNCs in both types of tonsils, which is not surprising, considering that they are the most abundant cells in secondary lymphoid organs. Of note, B lymphocytes were also the most frequently infected cells in PBMCs from acute COVID-19 adult patients (8). The median frequencies of CD8+ T lymphocytes in SARS-CoV-2-infected TMNCs were, respectively, 10% and 18% in palatine tonsils and adenoids, consistent with the rates found in PBMCs from acute COVID-19 patients (8). The infection of CD8+ T lymphocytes by viruses is surprising, considering that these are the very cells that perform cytotoxicity of virus-infected cells, and thus are central in the combat of viral infections. We have previously reported silent infection of tonsillar CD8+ T lymphocytes also by influenza A virus (27), suggesting that infection of these cells by viruses may have been previously overlooked. It has been suggested that SARS-CoV-2 persistence may be associated with a virus-specific CD8+ T cell response (28), which was not assessed in the present study.

SARS-CoV-2 was also detected in tonsillar CD14^+^ monocytes and CD123^+^ dendritic cells, which play important roles as components of the innate immune response. It is presently unknown whether SARS-CoV-2 in APCs results from virus antigen internalization or to their permissiveness to SARS-CoV-2 replication or both. Considering that monocytes, macrophages, and dendritic cells are infected by SARS-CoV-2 and that infection of human monocytes triggers inflammasomes (29), the infection of such cells in tonsils perhaps enhances inflammation in an already chronically inflamed tissue (29, 30).

At present, it is unknown whether SARS-CoV-2 infection of lymphocytes and APCs is detrimental to their function in secondary lymphoid tissues. Moreover, the antigenic specificities of infected lymphocytes in secondary lymphoid organs and whether some of these cells are naïve, memory, or innate cells are also presently unknown. With regard to the immune response in secondary lymphoid tissues, it has been shown that tonsils from COVID-19-convalescent children display persistent expansion of germinal centers and antiviral lymphocyte populations associated with interferon IFN-γ responses, evidence for persistent tissue-specific immunity in the upper respiratory tract of children after infection (31).

In the present study, the SARS-CoV-2 genome was detected by RT-qPCR in nasal washes from 5 of the 13 SARS-CoV-2-positive children, in the absence of COVID-19 symptoms, which is in general agreement with reports that up to 50% of children with SARS-CoV-2 infection may be asymptomatic (32). The asymptomatic shedding of SARS-CoV-2 in respiratory secretions from children with hypertrophic tonsils may be a source of virus in schools and households (33) and may also cause diagnostic confusion in ARI cases of other viral etiologies. Variable loads of SARS-CoV-2 RNA are shed in nasopharyngeal secretions and saliva from COVID-19 patients, including asymptomatic ones (34). In the present cohort, the median viral loads were not significantly different among different sampling sites.

In this study, we report that among 13 SARS-CoV-2-positive children, one child had a laboratory-confirmed SARS-CoV-2 infection approximately 5 months prior to tonsillectomy, suggesting that the patient either was experiencing a subacute infection or harbored persistent viral genomes within the tissues. This observation raises the possibility of prolonged or persistent genomic presence of SARS-CoV-2. Additionally, NSP-16 detection provides evidence of viral replication in at least half of the positive lymphoid tissues positive for SARS-CoV-2 by qRT-PCR, further supporting the notion of ongoing viral activity within these tissues. However, since we had no access to backup respiratory samples collected at the time of acute infection, it was not possible to ascertain whether the virus detected at the time of tonsillectomy of those two children was of the same strain causing the acute infection or a reinfecting new one. It has been shown also that SARS-CoV-2 proteins alter the host cell transcriptome, proteome, ubiquitinome, and phosphoproteome to evade host defenses and be able to persist in low-grade infection profiles (35).

Importantly, genome sequencing revealed SARS-CoV-2 of several Pangolin lineages in human tonsils, suggesting that tropism for tonsillar cells is not specific to certain lineages. Whole SARS-CoV-2 genome sequences were not obtained from the infected tissues, which is understandable, considering that the tissue samples may have undergone partial autolysis with viral RNA degradation. In addition, the heterogeneity in the intra-tissular distribution of SARS-CoV-2 RNA among different regions of the tonsils, which were randomly split for the different assays, may have also contributed to that. Nevertheless, the available coverage and sequence depth attained in 10 samples from eight patients enabled the safe calling of Pangolin lineages.

To the best of our knowledge, this study identifies sites of maintenance of RNA and protein in tissues in the upper respiratory tract of children. Besides epithelial cells, all major types of lymphomononuclear cells host SARS-CoV-2, which may contribute to the maintenance of SARS-CoV-2 RNA in lymphoid tissues of the upper respiratory tract, with still unknown potential immunoinflammatory consequences. These findings underpin the potential role of hypertrophic tonsils as sites of SARS-CoV-2 infection in children, for an undetermined prolonged time. Such smoldering SARS-CoV-2 infection might involve continuous low-level production of viral proteins and cell-to-cell transmission, which circumvent immune surveillance and subvert sterilizing immunity by low virus replication and possibly antigenic variation.

## SUPPLEMENTAL MATERIAL

Supplementary Figure Legends spectrum.01347-23-s0001.docx Legends for the supplementary figures.

Fig. S1 spectrum.01347-23-s0002.tif Control of immunohistochemistry.

Fig. S2 spectrum.01347-23-s0003.tif Expression of ACE2 and TMPRSS2 in SARS-CoV-2 infected tonsils.

Fig. S3 spectrum.01347-23-s0004.tif PileUp graphs of SARS-CoV-2 sequences.

Table S1 spectrum.01347-23-s0005.xlsx Spreadsheet with all patient's demographic information and test results.

ASM does not own the copyrights to Supplemental Material that may be linked to, or accessed through, an article. The authors have granted ASM a non-exclusive, world-wide license to publish the Supplemental Material files. Please contact the corresponding author directly for reuse.
