# Supplementary material for: Tonsils are major sites of persistence of SARS-CoV-2 in children
Source: Microbiol Spectr. 2023 Sep 22;11(5):e01347-23. doi: 10.1128/spectrum.01347-23 (PMC10581087; doi:10.1128/spectrum.01347-23)
Supplement: Supplementary Figure Legends — Legends for the supplementary figures. [file spectrum.01347-23-s0001.docx]

**Supplementary Figure 1:** Controls for Standardizing Immunohistochemistry Protocol for SARS-CoV-2 NP protein and SARS-CoV-2 NSP-16. A) Non-infected Vero CCL-81 cells served as a negative control for anti-NP staining, displaying no detectable signal. B) SARS-CoV-2-infected Vero CCL-81 cells exhibited a pronounced reddish signal when stained with anti-NP, indicating abundant presence of the NP protein. C) Non-infected Vero CCL-81 cells were used as a negative control for anti-NSP-16 staining, revealing no observable signal. D) SARS-CoV-2-infected Vero CCL-81 cells displayed multiple reddish signals upon anti-NSP-16 staining, suggesting the presence of several NSP-16 protein molecules. (magnification of 100 um).

**Supplementary Figure 2:** Expression of ACE2 and TMPRSS2 in tonsillar tissues. A). Palatine tonsil negative for SARS-CoV-2, showing signal for ACE2, TMPRSS2, and absence of signal for SARS-CoV-2 Spike. B) SARS-CoV-2 positive adenoids (AD) and palatine tonsils (PT), from different patients showing staining for ACE2, TMPRSS2, and SARS-CoV-2 Spike.

**Supplementary Figure 3:** Read and Coverage Reports of Pileup Analysis for all Sequenced Samples Positive for SARS-CoV-2. The figure provides results of the read length versus mean quality, read count by position, and the percentage of bases in the genome. (AD: adenoid; PT: palatine tonsil; NC: nasal cytobrush).
