# Supplementary figures and images for: Tonsils are major sites of persistence of SARS-CoV-2 in children
Source: Microbiol Spectr. 2023 Sep 22;11(5):e01347-23. doi: 10.1128/spectrum.01347-23 (PMC10581087; doi:10.1128/spectrum.01347-23)

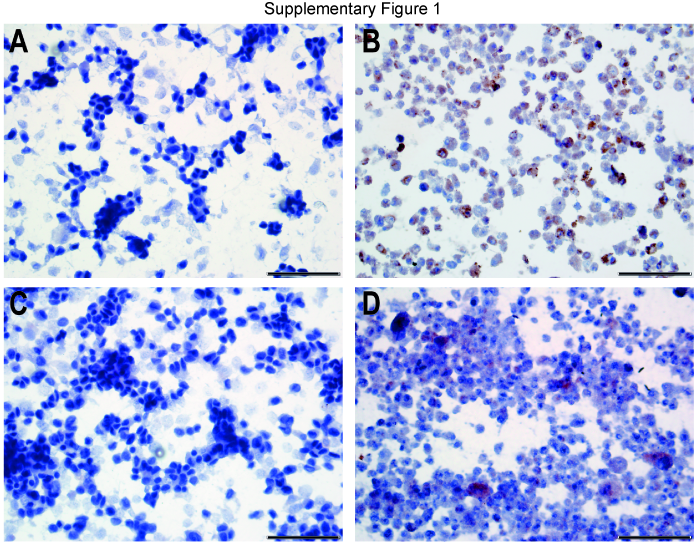

Supplement: Fig. S1 — Control of immunohistochemistry. [file spectrum.01347-23-s0002.tif]

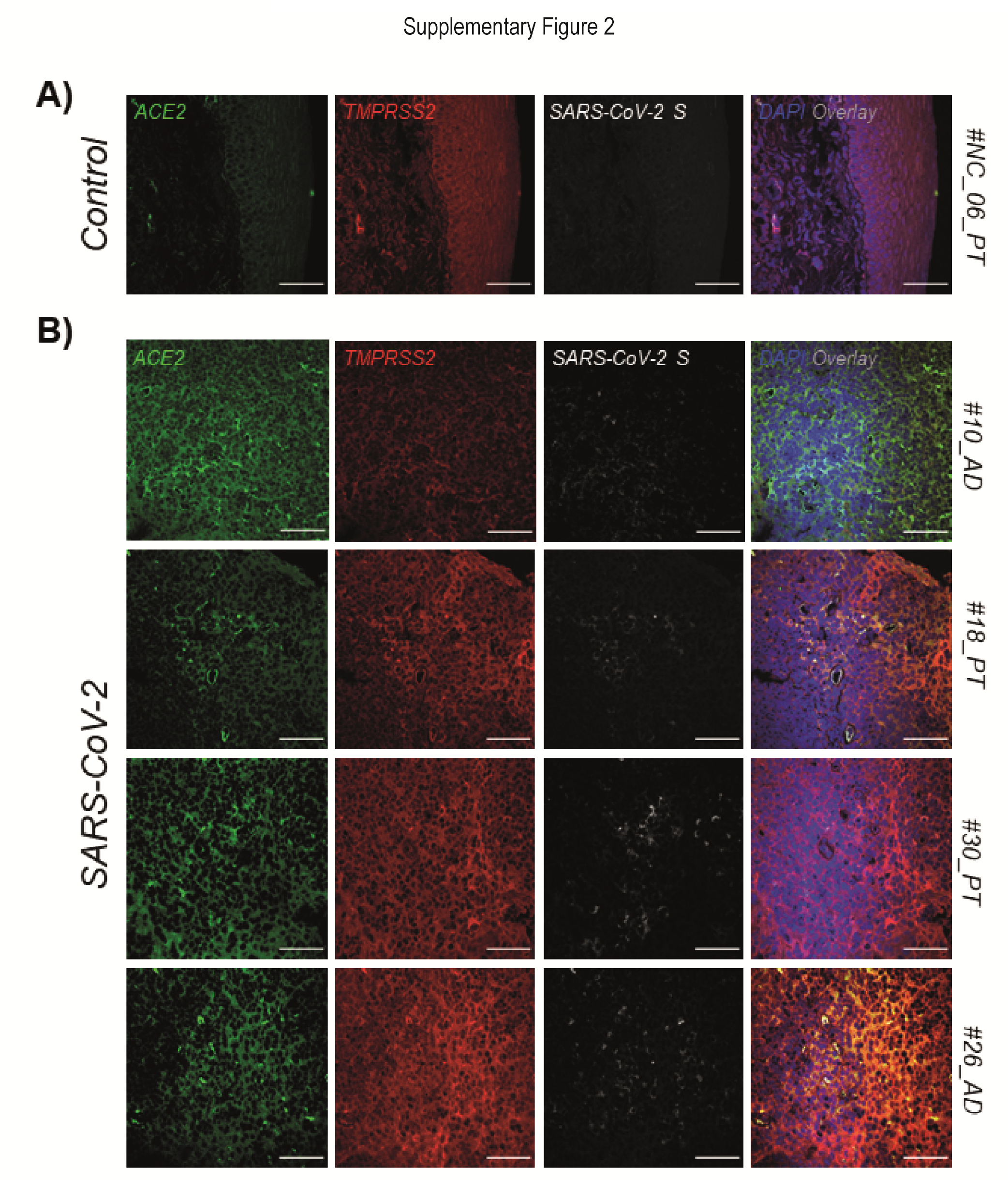

Supplement: Fig. S2 — Expression of ACE2 and TMPRSS2 in SARS-CoV-2 infected tonsils. [file spectrum.01347-23-s0003.tif]

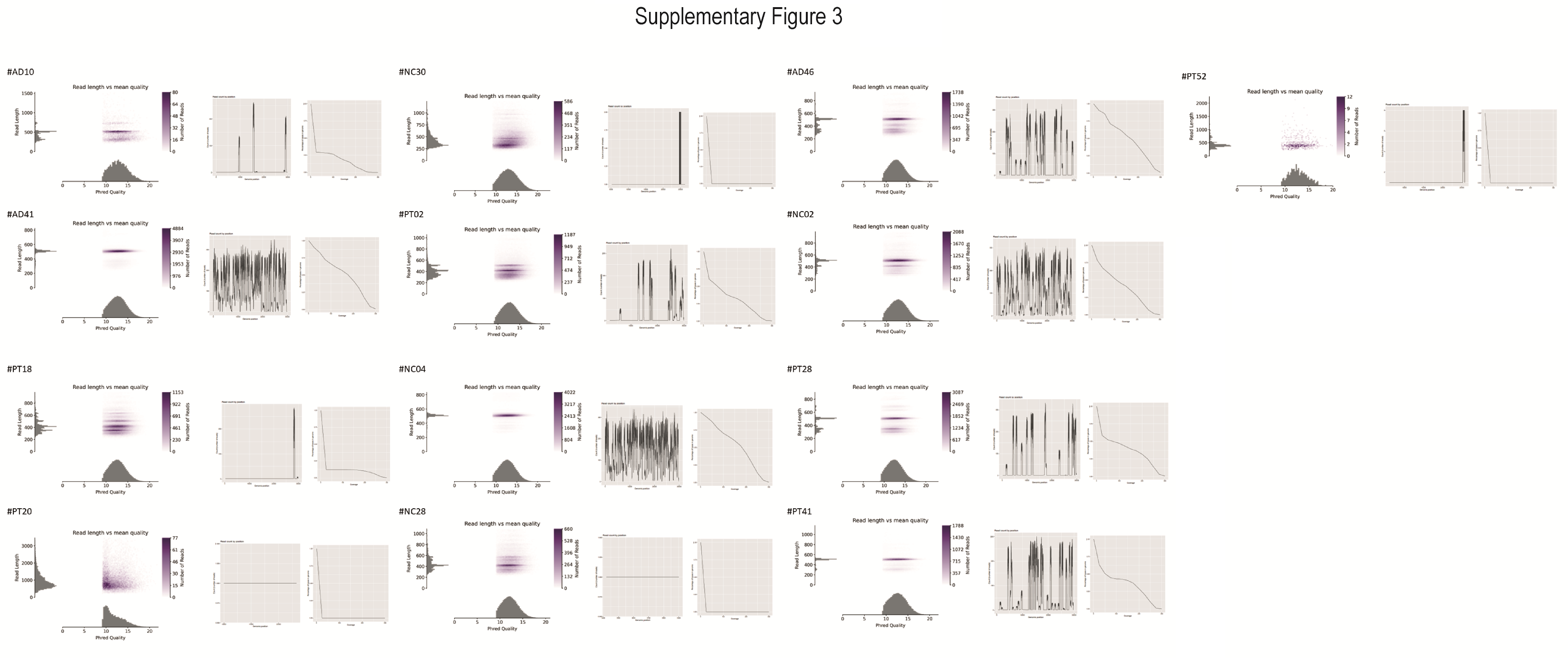

Supplement: Fig. S3 — PileUp graphs of SARS-CoV-2 sequences. [file spectrum.01347-23-s0004.tif]
